# Optimizing outcomes when treating functional neurological disorder in acute care settings: case reports depicting the value of diagnostic precision and timely and appropriate psychological interventions using an interdisciplinary framework

**DOI:** 10.3389/fpsyt.2024.1288828

**Published:** 2024-06-06

**Authors:** Melissa J. Greenfield, Aaron D. Fobian, Rachel E. Fargason, Badari Birur

**Affiliations:** Department of Psychiatry and Behavioral Neurobiology, University of Alabama, Birmingham, AL, United States

**Keywords:** functional neurological symptom, acute care, medical utilization, psychiatry, psychology, intervention, stigma

## Abstract

**Introduction:**

Unexplained physical signs and symptoms represent a significant portion of patient presentations in acute care settings. Even in cases where a patient presents with a known medical condition, functional or somatic symptoms may complicate the diagnostic and treatment processes and prognostic outcome. One umbrella category for neurologically related somatic symptoms, functional neurological disorder (FND), presents as involuntary neurological symptoms incompatible with another medical condition. Symptoms may include weakness and/or paralysis, movement disorders, non-epileptic seizures, speech or visual impairment, swallowing difficulty, sensory disturbances, or cognitive symptoms (1). While FND presents as neuropsychiatric, providers commonly report feeling hesitant to diagnose these disorders. Inexperience or lack of appropriate education on relevant research regarding evidence-based practices or standard of practice (SOP) may result in over- or underperforming diagnostic workups and consultations, utilizing inappropriate medications, and failing to offer evidence-based psychological interventions. Being mindful of these challenges when treating patients presenting with functional symptoms in acute care settings can help to support and protect the patients and care team and appropriately control healthcare costs.

**Methods:**

The University of Alabama at Birmingham Medical Center identified cases representing categories of quality and safety problems that arise in treating FND in acute care settings. Patients signed a consent form to participate in the case report. The case information for each was presented without identifying information.

**Discussion:**

The cases highlight potential challenges when caring for patients presenting with FND in acute care settings. The challenges covered include over- or underutilization of diagnostic workups and consultation, over- or underutilization of psychopharmacological medications, and over- or undertreating a medical condition when a functional symptom is present. In each case, these lapses and errors caused the patient distress, additional treatments, care delays, and delayed symptom remission. Additionally, these challenges have direct and indirect fiscal costs, which can be mitigated with the appropriate education and training, resources, and protocols. Hospitals can benefit from system-wide SOP to improve the identification and management of FND to prevent harm to patients. An SOP commonly presents to specific specialties and ensures the appropriate diagnostic workup, consultations, and timely evidence-based interventions.

## 1 Introduction

Up to one-third of patients across the lifespan who present in emergency and acute care hospital settings report functional symptoms that do not signify any physiological or medical condition (2). Up to 35% of patients in Epilepsy Monitoring Units are diagnosed with functional seizures (3). These patients typically present to primary care providers or other medical specialists first until the medical workup is exhausted (2). Providers are often uncertain in diagnosing and treating functional symptom presentations, resulting in variable treatment approaches that may unnecessarily place the patient at risk (4). Inadequate diagnosis and treatment of functional symptoms present a significant systemic health and financial burden due to unnecessary medical utilization and disability due to delays in appropriate treatment (5). Overall, patients with functional neurological disorder (FND) report decreased quality of life, similar to that of other neurological disorders. They spend significant time seeking an explanation and relief from their symptoms, often with little improvement in symptoms (6–9). Untreated FND symptoms can lead to alterations in ability and functional status, in addition to complicating secondary medical problems.

Previously known as conversion disorder, FND is characterized by one or more symptoms of altered voluntary motor or sensory function inconsistent with other neurological or medical conditions (1). Symptom presentations of FND include functional seizures or psychogenic non-epileptic seizures (PNESs), as well as motor (paralysis, weakness, gait disturbance, tremors, and tics), non-motor (altered awareness, abnormal sensation, pain, and fatigue), vision loss, and cognitive impairment (1, 10, 11). The symptoms result from errors in predictive coding; individuals develop automatic, reflexive, involuntary symptoms based on past experiences, allowing individuals to respond to sensory input more quickly (12, 13). It is important to note that patients with FND are not malingering (14, 15).

Research on FND has focused on understanding potential etiologies, comorbidities, and strategies for managing these symptoms. Studies have examined neuroimaging, other objective assessments, and neuropsychological and biopsychosocial mechanisms (16, 17, 18). Predisposing factors include a history of illness or physical injury in self or family members, increased bodily focus, other illness exposure, decreased sense of agency, and trauma (in adults but not children) or psychiatric symptoms (19, 20, 21, 9). FND presents unique diagnostic and treatment challenges with an equally debilitating impact on quality of life. Ideally, a patient would have a consistent multidisciplinary care team focused on internal medicine, neurology, and related medical specialties for medical comorbidities, psychiatry, psychology, and social work or case management. Currently, interdisciplinary treatment programs for FND are limited despite evidentiary support for maximizing outcomes.

FND presents a significant burden to the individual and healthcare system with both direct and indirect costs. For the individual, it may mean a change in ability status, income, and livelihood (22, 23). For the healthcare system, FND results in unnecessary costs and overutilization of resources, with an estimated total cost to the taxpayer of $1.2 billion related to hospital charges for all FND subtypes and all ages (5, 24). Due to a lack of diagnostic confidence, providers report difficulty definitively diagnosing FND and coding it as such in the medical records (25, 26). Studies have demonstrated that a definitive diagnosis and intervention for FND can reduce costs to healthcare systems by between 9% and 90.7% (5).

This article highlights the importance of diagnostic accuracy and appropriately addressing the medical and neuropsychiatric aspects of FND symptoms in acute care settings by leveraging consultation and interdisciplinary care. Appropriately setting a standard of practice (SOP) that meets ethical and practical guidelines and utilizing multispecialty and interprofessional teams can support the patient at the forefront of care, improving the prognostic outlook for all while reducing harm and expenditures.

## 2 Methods

Cases were identified after having been treated, in some cases, multiple times in the Emergency Room and then admitted to the University of Alabama at Birmingham Medical Center. The selected cases are considered representative of several categories of quality and safety problems that arise in treating FND in emergency and acute care settings. Patients signed a consent form to participate in the case reports. The information for each case is presented without identifying information.

## 3 Case reports

The following cases aim to highlight the types of potential challenges in identifying and treating FND in acute care settings when failing to leverage consultation and interdisciplinary teams.

### 3.1 Case 1: medical intervention may inadvertently exacerbate FND symptoms leading to prolonged symptom presentations complicating the hospital course and prognosis

Ms. R was a 27-year-old Caucasian woman with a history significant for a neurodevelopmental disorder, insulin-dependent diabetes controlled with an insulin pump, cutaneous lupus erythematosus, and diabetes insipidus. She had a craniopharyngioma resection at age 8, followed by the development of postoperative complications of pan-hypopituitarism and grand mal seizures, requiring prolonged hospitalization. She experienced an episode of status epilepticus requiring intubation and ventilation for 7 days at age 12. At age 10, she was also diagnosed with PNES, of a different character than her grand mal seizures, confirmed as non-epileptiform on electroencephalography (EEG). As an adult, the patient’s epileptiform seizures were under moderate control on lacosamide and levetiracetam, with seizures only occurring when the patient missed medication doses. The patient’s mother described her daughter as having a psychosomatic focus that occurred with any unpleasant stimuli, even mild discomfort, which was a typical trigger for a PNES event. During the index year of hospitalization described below, the patient’s conditions were overall stable; however, she presented to the emergency room (ER) approximately monthly, but occasionally up to two times in one week, with a variety of complaints including dehydration, shortness of breath, diarrhea, left lower quadrant pain, temporomandibular joint (TMJ) pain, and total body pain resulting in the following additional diagnoses: asthma, tachycardia syndrome [postural orthostatic tachycardia syndrome (POTS)], migraine, gastrointestinal reflux, gastrointestinal dysmotility, hypogammaglobinemia, and fibromyalgia. She was typically discharged from the emergency department (ED) with outpatient follow-up but admitted periodically and twice for surgical procedures (cholecystectomy and jaw surgery). On the most recent admission, Ms. R presented to the emergency department with dyspnea and concern for viral upper respiratory infection, which later evolved into concern about an allergic reaction to tree nuts. Other complaints included dysuria, headache, body aches, and abdominal pain. Emergency treatment included an anaphylaxis protocol of EpiPen and steroids to stabilize respiratory status.

Ms. R was subsequently admitted to the hospitalist service for further workup, which did not reveal any definitive cause for the dyspnea symptoms. The hospital course was complicated by medical codes for the new onset of seizure-like activity. Ms. R was observed to have rhythmic jerking, which subsided before any medical intervention. However, with continued hospitalization, the jerking returned for a prolonged period, and she was administered repeat doses of Ativan without clinical response. The neurology service was consulted, and non-rhythmic movements, irregular shaking, abdominal contractions, and bilateral fist clenching were reported to be consistent with PNES. Upon performing an emergent EEG, the neurology service found mild cerebral slowing consistent with mild cerebral dysfunction, with no epileptiform activity to correlate with observed typical episodes. The hospitalist service consulted the psychiatry and psychology services for further management of functional seizures.

The psychiatric service evaluated Ms. R utilizing a standardized medical and psychiatric history and mental status examination. Ms. R and her mother provided background information that revealed symptoms of mild depression and generalized anxiety, exacerbated in the hospital setting and in the context of her chronic medical conditions. Ms. R endorsed symptoms of post-traumatic stress disorder related to past medical experiences and childhood abuse. The evaluation also evidenced an increased pain response when presented with triggering stimuli. The psychiatrist recommended continuing escitalopram 20 mg daily, which had helped reduce dysthymia, and adding buspirone 5 mg bid to address the increased situational anxiety and underlying generalized anxiety disorder.

Next, the psychology service evaluated Ms. R and found that she had not received prior intervention for her functional seizures. Though the patient reported that the functional seizures were well-managed prior to her present hospitalization, she described her episodes as variable and triggered by sleep deprivation, overstimulation, illness, and pain. Her warning signs included calf and head pain and facial cooling, which progressed to loss of awareness or consciousness, visual and olfactory disturbances, and whole-body convulsions. The psychology service provided retraining and control therapy (ReACT), a standardized, research-supported cognitive behavioral treatment (CBT) intervention (27). In ReACT, an individualized treatment plan is developed using components of habit reversal, or the use of competing movements, to help the patient prevent or stop her functional seizures and retrain the symptoms from triggering. For example, her plan included a series of opposing responses when she experienced the warning signs to begin overriding the involuntary reflexes. She was also referred for inpatient physical and occupational therapies to build on her individualized treatment plan and target the jerking and convulsion.

Ms. R began using her treatment plan, and the episodes remitted quickly. The last episode she had was the night prior to the ReACT intervention, and after implementing her treatment plan, she remained episode-free until discharge 3 days later. Ms. R transitioned to the outpatient FND clinic, and when seen 4 weeks post-hospitalization, she continued to be episode-free. She reported that she had used her ReACT plan to successfully prevent functional seizures from starting and retrained the reflex to convulse. She was stabilized by regular treatments for 3 months in the outpatient psychology clinic and was discharged.

Upon a subsequent hospitalization for a pain workup, the patient remained on buspirone, which had reduced her overall anxiety levels, and escitalopram, which maintained a euthymic mood. She reported no PNES events since completing her therapy 5 months previously. However, she later alarmed her nurse with an episode of non-rhythmic shaking lasting 13 minutes. The primary medical team reconsulted the neurologist, who, after obtaining a history from the patient’s mother, recommended the following: 1) avoiding lorazepam as unhelpful for similar events and with potential for respiratory suppression; 2) not to repeat the extended EEG, as family believed it made the patient’s anxiety worse; and finally 3) use of diphenhydramine and/or hydroxyzine for future PNES events, as these had helped in the past.

### 3.2 Case 2: utilizing psychotropic medications as the sole intervention to treat FND symptoms may lead to altered mental status and delay treatment progress from other interdisciplinary treatments

Ms. T was a 49-year-old black woman with a history of functional seizures, chronic pain, asthma, migraine, and bipolar disorder. She had five previous visits to UAB ER, mostly related to syncopal events. A workup in the EEG laboratory determined that FND was present, as she had no evidence of epileptiform activity during her typical events, including loss of consciousness, will-related falls, staring, and unresponsiveness. She also had a childhood history of trauma, motor vehicle accident (MVA) in 2010 with resultant paralysis for 2 years, and lifelong chronic medical conditions supporting the diagnosis of FND. The patient had multiple CT and magnetic resonance imaging (MRI) scans, EEGs, and angiograms in the course of these workups with normal results. On the index hospitalization, Ms. T presented to the emergency department with altered mental status, aphasia, and paralysis of the upper and lower extremities. Emergency code stroke protocol was implemented, including examination, laboratory tests, and imaging of the head and neck. The imaging was without acute abnormality and showed no large vessel occlusion or perfusion without ischemia. The laboratory test values were within normal limits. Diagnostically, without the presence of localized neurological examination findings and with reassuring imaging, Ms. T was not considered a candidate for thrombolysis or thrombectomy. However, encephalopathy was considered in the differential diagnosis. Therefore, Ms. T was to be evaluated further for stroke with additional imaging. The primary team noted concern for prior events with similar presentations.

Ms. T was then admitted to the neurology service for further diagnostic workup, where she remained non-verbal and immobile with limited awareness and initiation. After a few days, Ms. T became increasingly coherent; she was observed to groan and utter unintelligible words, with increasing responsiveness and interaction upon examination. Ms. T was also observed to have increased spontaneous movement of her upper extremities. Her family at bedside reported a similar presentation in 2021, at which time she was diagnosed with functional seizures following a normal EEG, which the patient referred to as a “stroke”. Relatedly, her family reported headaches triggered her episodes, and Ms. T reported having a headache beginning before her current episode, which continued into her hospitalization.

The neurology service ordered a repeat long-term EEG, MRI, with and without contrast, lipid panel, thyroid, vitamin B12, folate, rapid plasma reagin (RPR), quantitative detection of human immunodeficiency virus (HIV), and nutritional workup. All imaging and laboratory test results returned normal. Results of the long-term EEG found no epileptiform discharges during captured typical events, confirming the diagnosis of functional seizures. The neurology service had continued her reported at-home medications of topiramate, fluticasone propionate/salmeterol inhaled, albuterol, lithium (600 mg), valproic acid (1,000 mg), lamotrigine (200 mg), quetiapine (300 mg bid), pregabalin (300 mg), oxycodone with acetaminophen, and gabapentin. However, the neurology service discontinued triptans, ibuprofen, and lidocaine patch. The neurology service consulted with the psychology service at this time.

The psychology service evaluated Ms. T, who was somnolent and minimally responsive. She confirmed similar episodes since 2021, which began with a headache and progressed to facial numbness and tingling, followed by speech arrest and eventually full-body paralysis. Ms. T denied prior treatment for her episodes. At this point, she was utilizing her upper extremities, and her speech had improved. She denied the ability to use her lower extremities. The consulting psychologist (MG) implemented the ReACT intervention, developing a retraining plan that included a series of opposing responses once she felt the warning sign of facial numbness and tingling. Her treatment plan began with a facial massage, followed by sensory orientation and oral, upper, and lower extremity motor movements. She was encouraged to use her treatment plan to retrain the involuntary reflexes and avoid assistance from others or devices to unpair the involuntary response. She was referred for inpatient physical and occupational therapies to target the overall weakness, paralysis, and mild aphasia and avoid deconditioning.

For approximately 5 days, despite the patient using the ReACT intervention and engaging in therapies, she made minimal progress and continued to be somnolent and lethargic. Given her lack of sufficient progress and inability to adequately engage in activities of daily living (ADLs), the patient was slated to be discharged to a skilled nursing facility. However, the neurology and psychology services discussed possible etiologies for the somnolence. They determined it was due to a medication side effect, which was impacting her ability to engage in the treatment plan and therapies. The patient’s family at the bedside confirmed that the patient’s increased somnolence was likely due to quetiapine, which had the same effect in the past and had been discontinued for this reason. The psychology service recommended a consultation with psychiatry for medication management.

The psychiatry service evaluated the patient utilizing a standardized medical and psychiatric history and mental status examination. The evaluation revealed bipolar disorder with a mild depressive phase. No addition was made to the medication recommendations, as the psychiatry service believed the low mood was situational. The psychiatry service recommended checking valproic acid levels prior to the patient leaving the hospital. They concurred with a functional basis for Ms. T’s current presenting symptomology. They also confirmed the patient had adverse side effects on quetiapine in the past and that the medication had been previously discontinued prior to her hospitalization. Subsequently, quetiapine was discontinued by the neurology service.

Two days after discontinuing quetiapine, Ms. T endorsed significant improvements in her mentation. Once she was able to remain awake and engaged in the treatment plan and therapies, she improved significantly over the span of 1 week. Ms. T’s speech had returned, and she regained all functionality of her body. She was discharged home 2 days later, fully functional and performing activities of daily living and most instrumental activities of daily living, except for driving. Once discharged, Ms. T began treatment in the outpatient FND clinic. She endorsed the successful utilization of her treatment plan to retrain episodes with an overall reduction in her functional seizure rate and severity. Ms. T was discharged from treatment upon successful remittance of her functional symptoms.

### 3.3 Case 3: prolonged failure to directly treat worsening FND-related excessive motor movements led to life-threatening rhabdomyolysis followed by severe secondary medical complications and limited psychosocial functioning

Mr. G was a 20-year-old Caucasian man with a history of Tourette’s syndrome, FND, obsessive-compulsive disorder (OCD), anxiety, panic attacks, depression, and post-traumatic stress disorder (PTSD). He was diagnosed with Tourette’s syndrome and tic disorders at age 6, with symptoms including high-pitched shrieking and facial grimacing. He also had compulsions to bite handheld objects. In middle school, his tics worsened, and ballistic movements developed with an expanding verbal component. During high school, his movements were affecting his daily functioning. He had previously been treated in his mid-western hometown with multiple failed trials of psychopharmacologic agents to target mood symptoms with no reduction in motor symptoms. Mr. G was admitted to the local Children’s Hospital in July 2020 for continuous daily symptoms with the onset of seizure-like episodes, including whole-body convulsions. He was diagnosed with functional seizures based on a normal long-term video EEG showing no epileptiform correlates. His care team at the time proceeded with the implantation of a deep brain stimulator (DBS) to address his impaired continuous motor movements. His large and small motor symptoms had not improved 3 months postoperatively. However, his mood improved with therapy and psychopharmacologic treatment targeting OCD and anxiety symptoms. By 6 months postoperatively, Mr. G’s continuous jerking motor symptoms worsened to the point that he was readmitted to the hospital for rhabdomyolysis. He subsequently incurred a series of medical complications, including aspiration pneumonia, respiratory failure, tracheostomy, and thrombophlebitis of a peripherally inserted central catheter (PICC) line. Cranial CT scan on January 20, 2023 was reported to be normal. Mr. G remained in a coma for 7 days, and as a result, he lost significant weight, muscle tone, physical strength, and stamina, for which he required physical rehabilitation. After discharge, Mr. G’s symptoms were less intense but continued. He attended an intensive cognitive behavior program for OCD and anxiety, which the family stated “calmed him down”; however, benefits desisted after discharge from the program. Mr. G. later left college due to persistent motor symptoms. He experienced daily muscle soreness, and while able to provide basic self-care, he is unable to safely use a knife, drive, or hold a job and is dysphoric over loneliness and limited lifestyle. In this context, Mr. G and his family sought a multidisciplinary FND-specific treatment program.

Mr. G presented to our intensive outpatient program (IOP) as an out-of-state participant for a time-limited treatment protocol to address his long-standing symptoms of FND. The team neurologist observed him to have frequent involuntary non-linguistic abrupt vocalizations, truncal lurching, tongue protrusions, abrupt neck flexions, occasional abrupt shoulder elevations, and episodic ballistic arm raising followed by rapid downward movement of arms. These movements were notably suppressed with distraction maneuvers, such as being told to discard his chewing gum. Gait and motor strength were normal. The psychology team noted during the initial interview that he had stopped biting himself but displayed repetitive vocal tics and hit himself repeatedly while “completing a motor tic until it felt right”. His parents reported that these were absent during sleep. He also experienced approximately one panic attack per day, which was an improvement over four to five per day in December 2022, and his parents reported that he was re-experiencing and vigilance due to medical trauma during one of his hospitalizations.

He also displayed frequent vocal tics throughout the psychologist evaluation, consisting of grunting, throat clearing, and occasionally whining. The longest time between vocal tics was 20 seconds. Motor tics noted during the psychologist’s interview included repositioning in his chair, hitting his foot against his legs, and rapid flinging arm movements. He also hit his head a few times during the 1-hour session. The longest time between motor tics was 25 seconds. Current medications included the following: buprenorphine 4 mg tid, gabapentin 600 mg bid, clonazepam 0.25 mg bid, guanfacine 2 mg bid, methocarbamol 100 mg tid, quetiapine 125 mg daily, and sertraline 200 mg daily. The neurologist diagnosed functional neurological symptom disorder (FNSD) with mixed symptoms and no other neurologic disorder but opted not to change medications until the therapy was completed.

Mr. G was treated at the UAB Interdisciplinary FND Treatment Program during a 2-week intensive outpatient program, including psychology, physical therapy (PT), occupational therapy (OT), and speech therapy (ST). Psychoeducation was provided to the patient and his caregivers regarding FNSD and its treatment. His parents were instructed not to intervene except to maintain safety and to prompt the patient to “do his plan”. A retraining plan was developed, which included calming and controlling thoughts, as well as opposing responses for motor symptoms such as making intentional circles with arms and legs in the air and humming or tapping a musical type beat with his mouth. He was instructed to complete opposing responses whenever he felt a tic-like behavior may start or disrupt an escalation. In addition to intensive retraining work during sessions provided, the patient and parent treatment plans were implemented for continual use in response to all symptoms. He was encouraged to engage in activities of daily living, daily exercise, and social activities of interest.

Mr. G concurrently engaged in outpatient PT, OT, and ST. The psychology service continued sessions with Mr. G two times per week to reinforce the use of the comprehensive treatment plan and make necessary adjustments. Over the 2-week period, the patient, family, and treating team observed an overall reduction in motor and vocal symptom occurrences, duration, and severity, with a better ability to control symptoms completely for intervals of time. The patient was then discharged home to continue this intervention with his local therapist and local OT, PT, and medical providers.

## 4 Discussion

Patients with FND who are seen in emergency settings or admitted to the hospital can present with functional complaints superimposed on complex medical and psychiatric diagnoses that confound treatment planning and do not improve until adequately diagnosed and treated. Furthermore, the attention given to physical symptoms in the hospital environment can inadvertently exacerbate FND symptoms, leading to a cycle of increasing workups and interventions that increase the patient’s somatic anxiety and the possibility of iatrogenic harm. Common challenges across the medical setting represented by the cases above appear in three general categories.

### 4.1 Case 1: overperforming a diagnostic workup or repeating previously normal workups without indication

The case of Ms. R demonstrates how undergoing a repeat extensive and expensive diagnostic workup may negatively impact the hospital course and prognosis. Although some providers are hesitant to make a diagnosis of FND due to fear that they have missed something, misdiagnosis of FND is rare, with research showing the rate of misdiagnosis has consistently been approximately only 4% since 1970 (28). Having the patient undergo unnecessary invasive testing, imaging, laboratory tests, or exploratory surgeries may delay appropriate interventions to target the symptoms for remission. This may also confound the medical picture by reinforcing symptoms via attention to or reinforcing a “sick role”. Additionally, these tests and procedures may cause iatrogenic complications. An appropriate targeted workup is best practice, as it considers inconsistent medical symptoms and complaints, given this population’s high rates of comorbidities (29). Overall, providers may increase confidence in their diagnostic workup and definitively diagnose FND if they approach the condition as a diagnosis of inclusion versus exclusion (30). Clear documentation of the FND during the index hospitalization helped prevent the automatic implementation of seizure workup protocols. Instead, the neurologist was able to guide the primary team in addressing the FND more specifically and appropriately.

### 4.2 Case 2: overuse of medication for symptoms/episodes that are FND-related may result in adverse outcomes, prolonged symptoms, and reduced functionality

The case of Ms. T demonstrates how prescribing unwarranted psychotropic medications can result in unwanted side effects or prolonged symptom presentation. Research demonstrates medications do not reduce functional seizure symptoms (31, 32). Fortunately, Ms. T was able to have significant symptom improvement and avoid nursing home placement with successful consultation and interdisciplinary teamwork. A common analogous example is a patient who is prescribed a benzodiazepine, with inherent habituation, tolerance, and withdrawal risks, to try to reduce the occurrence of functional seizures. The use of benzodiazepines by emergency medical personnel for FND symptoms has been found to sometimes result in critical sedation or intubation (33). Unwarranted use of medications for FND symptoms also serves to reinforce that the symptoms are “in their head” and that they do not have control, thereby potentially thwarting rehabilitation intervention efforts aimed at symptom remission.

### 4.3 Case 3: failure to provide appropriate and timely diagnosis and interventions for both functional symptoms and other medical conditions in the presence of functional symptoms

As in the case of Mr. G, this may also lead to a patient undergoing an invasive procedure that is not evidence-based for the treatment of FND. Another example is a patient being denied a dental procedure due to a prior diagnosis of FND. A prior diagnosis of FND should not impact the SOP for evidence-based care over concerns regarding the validity of their symptom presentation. Overall, appropriate competencies in FND treatment and available consultation and multidisciplinary interventions for patients would serve to improve prognostic outcomes.

Studies have found that general psychotherapeutic interventions increase overall life satisfaction in both the presence and absence of psychiatric history or symptoms, though significantly, they do not improve FND symptoms (34, 35, 36). Historically, FND has carried a stigma. This has typically stemmed from the overreliance on diagnostic tools that are not sensitive and specific enough to capture all symptoms resulting from abnormal functioning of the mind-body. Medical providers may consider increasing their comfort in discussing FND with patients to ensure treatment compliance by reducing the stigma associated with such diagnoses. When discussing these diagnoses with patients, it may be helpful to posit them in the framework of errors in the predictive coding of experiences that result in automatic involuntary reflexes. Basic biological survival mechanisms embedded in the neural networks attempt to predict incoming stimuli to increase the likelihood of survival but may make errors (12, 13). For example, common daily experiences may trigger a physical response interpreted as a serious medical condition. With repeated exposure to the trigger, the physical response may continue to occur until it is retrained (ReACT; 27). The good news is that research suggests that FND may be treatable without any invasive medical interventions. Several studies have demonstrated the potential efficacy of CBT and rehabilitative approaches (27, 37, 36). In conjunction with a multimodal treatment approach to symptoms, an evidence-based protocol can help a patient gain functionality toward overall health and wellness. The current standard of care for FND psychosocial management consists of patient and family education, evidence-based psychotherapy, and physical rehabilitation. A review of evidence-based psychotherapies is beyond the scope of this article, and the reader is referred to a review by Onofrj et al. (38). Patients must be validated for the existence of a true condition and the possibility of reversal if they adhere to appropriate treatments. The therapy will then be tailored to the patient’s individual needs and symptoms as described in the patient’s case. Recognition of key stressors and therapy to teach self-calming techniques are key components of these treatments.

Limitations to the present paper include the number of cases presented and limited research available on interdisciplinary FND treatment teams in acute care settings. Additionally, we cannot compare the treatment provided at UAB to other treatments provided for FND. Case reports inherently lack control comparisons to compare outcomes. Another limitation, particularly brought out by Case 1, is the subjectivity of symptom presentation in many medical conditions and how, without objective measures, these can be confounded with medical conditions. Additionally, treatment of the FND is just the tip of the iceberg in addressing a complex patient with cognitive differences and somatic symptom disorder. To this day, it is challenging to verify which of Ms. R’s surgeries were necessary or helpful and which medical diagnoses were accurate. However, the clear documentation of FND at the index hospitalization set the model going forward for other specialists providing care to this patient to make their documentation clearer: the patient’s rheumatologist, for example, documented cutaneous systemic lupus (documented by biopsy and pathology). Controlled trials in the acute care setting are needed. The primary goal of this report is to alert the medical community to this phenomenon, which is under-recognized and has not been documented.

## 5 Conclusion

Potential harm when treating FND in acute care settings includes errors and delays of care that can occur due to general clinical inexperience, complicated medical comorbidities, and failure to maximize consultation and appropriate interventions with this patient population. An interdisciplinary approach is the key to successful diagnosis and treatment in cases of FND. Providers treating patients with FND should practice careful communication with consulting services to successfully provide appropriate psychoeducation, develop and implement evidence-based treatment plans, and transfer to interdisciplinary outpatient services and supports.

## Data availability statement

The original contributions presented in the study are included in the article/supplementary material. Further inquiries can be directed to the corresponding author.

## Ethics statement

Written informed consent was obtained from the individual(s) for the publication of any potentially identifiable images or data included in this article.

## Author contributions

MG: Conceptualization, Formal analysis, Methodology, Validation, Writing – original draft. AF: Conceptualization, Formal analysis, Methodology, Supervision, Validation, Writing – review & editing. RF: Conceptualization, Methodology, Resources, Supervision, Validation, Writing – review & editing. BB: Conceptualization, Formal analysis, Supervision, Validation, Writing – review & editing.
